# A multicenter noninferior randomized controlled study of sentinel lymph node biopsy alone versus sentinel lymph node biopsy plus lymphadenectomy for patients with stage I endometrial cancer, INSEC trial concept

**DOI:** 10.1186/s12885-023-11226-1

**Published:** 2023-12-02

**Authors:** Yanglong Guo, Lu Sun, Xi Chen, Qiang Wen, Zhuyan Shao, Xuedong Tang, XiaoJun Shi, Jinyu Wang, Yingli Zhang, Tao Zhu

**Affiliations:** 1 Department of Gynecologic Oncology, Zhejiang Cancer Hospital, Hangzhou Institute of Medicine (HIM), Chinese Academy of Sciences, Hangzhou, Zhejiang 310022 China; 2 Department of Gynecologic Oncology, Jiaxing Maternity and Child Health Care Hospital, Jiaxing, Zhejiang People’s Republic of China; 3 Department of Gynecologic Oncology, The First Hospital of Jiaxing, Affiliated Hospital of Jiaxing University, Jiaxing, Zhejiang People’s Republic of China; 4 Department of Medical Records Statistics, Zhejiang Cancer Hospital, Hangzhou Institute of Medicine (HIM), Chinese Academy of Sciences, Hangzhou, Zhejiang 310022 China

**Keywords:** Endometrial cancer, Sentinel lymph node, Indocyanine green, Near-infrared fluorescence imaging, Lymphadenectomy

## Abstract

**Background:**

Up to the present time, there has remained a lack of strong evidence as to whether sentinel lymph node biopsy can replace lymphadenectomy for early endometrial cancer. The traditional surgery for endometrial cancer includes pelvic lymphadenectomy and paraaortic lymph node resection, but complications often seriously affect patients’ quality of life. Two randomized controlled trials with large samples have proved that lymphadenectomy does not improve the overall recurrence rate and survival rate of patients. On the contrary, it increases the incidence of complications and even mortality. The current trial is designed to clarify whether sentinel lymph node biopsy can replace lymphadenectomy for early endometrial cancer patients with negative lymph nodes.

**Methods:**

This study is a randomized, open-label, multicenter and non-inferiority controlled clinical trial in China. Potential participants will be patients with pathologically confirmed endometrial cancer at the Zhejiang Cancer Hospital, Jiaxing Maternity and Child Health Care Hospital, and the First Hospital of Jiaxing in China. The total sample size for this study is 722.

Patients will be randomly assigned in a 1:1 ratio to two groups. Patients in one group will undergo sentinel lymph node biopsy + total hysterectomy + bilateral salpingo-oophorectomy ± paraaortic lymph node resection. Patients in the other group will undergo sentinel lymph node biopsy + total hysterectomy + bilateral salpingo-oophorectomy + pelvic lymphadenectomy ± paraaortic lymph node resection. The 3-year disease-free survival rate, overall survival rate, quality of life (use EORTC QLQ-C30 + QLQ-CX24), and perioperative related indexes of the two groups will be compared.

**Results:**

We expect to find that for patients with early endometrial cancer, the 3-year disease-free survival rate following sentinel lymph node biopsy with indocyanine green combined with near-infrared fluorescence imaging is similar to that following lymphadenectomy. The operation time, as well as incidence of pelvic lymphocyst, lymphedema of lower limb, and edema of vulva in patients who only undergo sentinel lymph node biopsy are expected to be significantly lower than in patients who undergo lymphadenectomy. The quality of life of patients who undergo sentinel lymph node biopsy alone will be significantly better than that of patients who undergo lymph node dissection.

**Conclusion:**

This will prove that the prognosis of sentinel lymph node biopsy alone with indocyanine green combined with near-infrared fluorescence imaging is not inferior to that of sentinel lymph node biopsy plus lymphadenectomy for early stage endometrial cancer with negative nodal assessment intraoperatively. In addition, sentinel lymph node biopsy alone with indocyanine green combined with near-infrared fluorescence imaging results in fewer surgical complications and gives patients better quality of life.

**Trial registration:**

chictr.org.cn, ChiCTR1900023161. Registered 14 May 2019, http://www.chictr.org.cn/edit.aspx?pid=38659&htm=4.

## Background

Endometrial cancer (EC) is one of the most common gynecological malignancies. The incidence rate is increasing each year. Lymph node metastasis is the main factor affecting prognosis while nodal status is extremely important in guiding adjuvant therapies [1–3]. The 5-year survival rates of patients with stage I non-metastatic endometrial cancer and patients with pelvic or paraaortic lymph node involvement are 90% and 44% ~ 52%, respectively [4].

The sentinel lymph node (SLN) is the first lymph node that a primary solid tumor must pass through when lymph node metastasis occurs. It reflects the state of lymph node metastasis in the whole region [5]. Indocyanine green (ICG) combined with near-infrared fluorescence imaging can accurately locate the sentinel lymph nodes of patients with early endometrial cancer, and then sentinel lymph node biopsy can be performed during the operation. If the frozen pathology is negative, the patient does not need lymphadenectomy. In addition, ICG combined with near-infrared fluorescence imaging has been proved to be superior to ^99^Tc, blue dye and other methods, especially for obese patients [6–9]. Most patients with endometrial cancer are obese, so this technology is very suitable for patients with early endometrial cancer.

Lymph node metastasis is the main type of metastasis in endometrial cancer. The evaluation of lymph node status and the selection of surgical methods are of great significance when formulating a treatment plan and predicting a prognosis. The traditional surgery for endometrial cancer includes pelvic lymphadenectomy and paraaortic lymph node resection, but complications often seriously affect patients’ quality of life. Two randomized controlled trials with large samples have proved that lymphadenectomy does not improve the overall recurrence rate and survival rate of patients. On the contrary, it increases the incidence of complications and even mortality [1, 2]. However, inclusion of large number of patients with low-grade, low stage disease potentially diluting the benefit of lymphadenectomy may be the limitations of the two trials. Extensive lymphadenectomy has significantly increased complications.

So far, there has been no consensus on whether sentinel lymph node biopsy can replace lymphadenectomy in the treatment of patients with early endometrial cancer. The following are the results of sentinel lymph node studies to date. In 2017, Lancet Oncology published the results of robotic sentinel lymph node biopsy (FIRES study) in patients with stage I endometrial cancer. The results showed that the sensitivity of sentinel lymph node biopsy in the diagnosis of lymph node metastasis was 97.2%, and the negative predictive value was 99.6% [10]. Some scholars express concern that sentinel lymph node mapping will miss isolated paraaortic lymph nodes, especially in patients with a high metastasis rate of paraaortic lymph nodes (such as high-grade and tumor-infiltrating deep muscle layer) [11]. To address this concern, Bogani et al. [12] conducted a meta-analysis of the clinical data of 3,536 patients with endometrial cancer who underwent sentinel lymph node biopsy (1,249 cases) or lymphadenectomy (2,287 cases). The results showed that the positive detection rate of pelvic lymph nodes in the sentinel lymph node mapping group was higher than that in the lymphadenectomy group (14.7%: 9.9%). There was no significant difference in the positive detection rate of paraaortic lymph nodes between the two groups. Also, there was no significant difference in the total recurrence rate (4.3%: 7.3%) or lymph node recurrence rate (1.2%: 1.7%) between the sentinel lymph node mapping group and the lymphadenectomy group. In addition, a study found that the sensitivity and negative predictive value of sentinel lymph node biopsy in patients with high-risk histological types of endometrial cancer were 95.0% and 98.6%, respectively. 89% of patients had at least one sentinel lymph node mapping. Of these, 58% were bilateral sentinel lymph nodes, 40% were unilateral sentinel lymph nodes, and 2% were isolated paraaortic lymph nodes [13]. Maria C. Cusimano and Sarah E. Ferguson underwent SENTOR trial and found the acceptable accuracy of sentinel lymph node mapping in high-grade endometrial cancer. Of 27 patients with nodal metastasis, sentinel lymph node mapping correctly identified 26 of them (96% sensitivity; 95% CI; 81%–100%) [14]. A study jointly carried out by Memorial Sloan-Kettering Cancer Center and the Mayo Clinic found that compared with lymphadenectomy, sentinel lymph node biopsy for endometrial cancer with deep myometrial invasion did not affect the tumor outcome, and the prognosis of lymph-node-negative patients was better, but this had nothing to do with the evaluation method of lymph nodes [15]. Sentinel lymph node mapping did not affect the prognosis of high-risk endometrial cancer [16]. Moreover, How et al. [17] systematically evaluated 5,348 patients in 48 studies. The results showed that the total detection rate of SLN detected by ICG combined with near-infrared fluorescence imaging was 94%, which was significantly higher than that by blue staining (86%) and radionuclide method (86%). 34 studies showed a total sensitivity of 94% and a negative prediction rate of 100%. Therefore, indocyanine green combined with near-infrared fluorescence imaging technology has a good clinical application prospect.

## Methods

### Trial design and surgical quality assurance

**Fig. 1:**
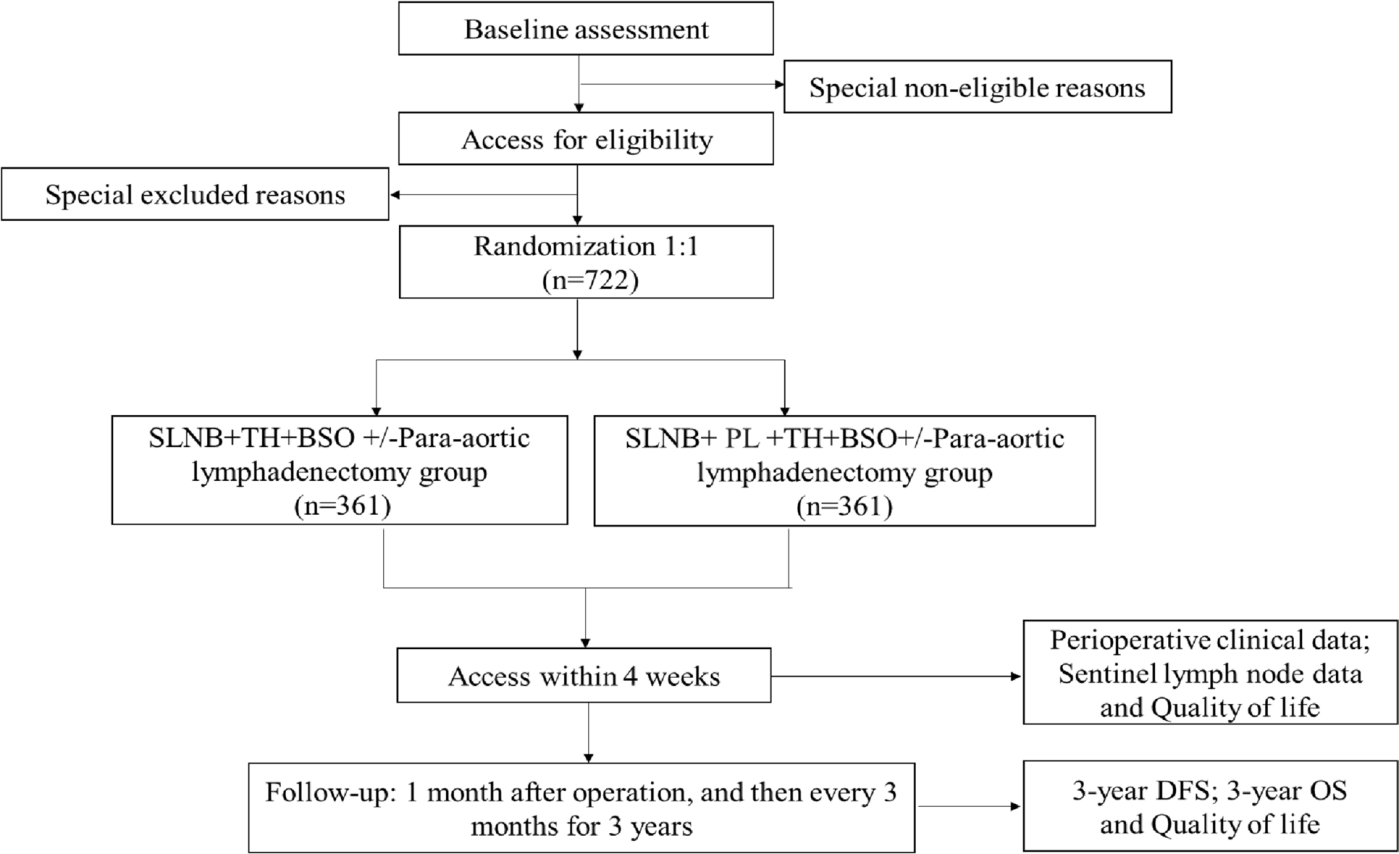
Study flow chart. SLNB: Sentinel lymph node biopsy; TH: Total hysterectomy; BSO: Bilateral salpingo-oophorectomy; PL: Pelvic lymphadenectomy; DFS: Disease-free survival; OS: Overall survival

The INSEC study (ChiCTR1900023161) is a randomized, open-label, multicenter and non-inferiority controlled clinical trial in China. Potential participants will be patients with pathologically confirmed endometrial cancer at the Zhejiang Cancer Hospital, Jiaxing Maternity and Child Health Care Hospital, and the First Hospital of Jiaxing in China. Patients will be randomly assigned in a 1:1 ratio to two groups, Arm A and Arm B (Fig. 1). Patients in Arm A will undergo sentinel lymph node biopsy (SLNB) + total hysterectomy (TH) + bilateral salpingo-oophorectomy (BSO) ± paraaortic lymph node resection. Patients in Arm B will undergo SLNB + TH + BSO + pelvic lymphadenectomy (PL) ± paraaortic lymph node resection.

### Qualification of surgeons

So far, there is no consensus on whether sentinel lymph node biopsy can replace lymphadenectomy in the treatment of patients with early endometrial cancer. The need for qualification and training of surgeons involved in these studies is particularly important, especially on minimally invasive surgery techniques and cervical ICG injection. The current study asks that surgeons involved in the study have operated on more than 50 cases of endometrial cancer by independent minimally invasive surgery and injected ICG into the cervix in more than 30 cases. In addition, it is suggested that the participating center should be a gynecological oncology center with sufficient patients, and should have sentinel lymph node ultra-staging technology. Then, it will be carefully evaluated in stratification. We look forward to the prognostic data of the study in a few years.

### Inclusion criteria

1. Age is 18–70
2. Endometrial diagnostic curettage pathology suggests endometrioid adenocarcinoma (including cancerization)
3. Pelvic magnetic resonance imaging (MRI) shows that the lesion is limited to the uterine body
4. Gynecological examination: uterus less than 2 months pregnant (length ≤ 10 cm, width ≤ 8 cm)
5. No suspicious nodes on abdominal computed tomography (CT), according to Response Evaluation Criteria in Solid Tumours (RECIST) 1.1
6. Performance Status-Eastern Cooperative Oncology Group (PS-ECOG) 0–1 points
7. Blood routine and biochemical examination: leukocyte ≥ 4000 cells mm3, platelet ≥ 10000/mm3, hemoglobin ≥ 8.0 GM/dl, serum creatinine ≤ 1.3 mg/dl, serum urea nitrogen ≤ 1.5 mg/dl
8. Patient has the ability to sign informed consent and comply with follow-up actions
9. Only patients undergoing minimally invasive surgery using ICG combined with near-infrared fluorescence imaging are included.

### Exclusion criteria

1. Pathology suggests non-endometrioid adenocarcinoma
2. Pregnancy or lactation
3. Patients with synchronous cancer
4. Received neoadjuvant chemotherapy and/or radiotherapy
5. Allergic reaction to ICG
6. Severe cardiopulmonary vascular disease leading to intolerance to surgery
7. Mental illness
8. Patient does not agree to sign informed consent
9. Cervical stroma invasion
10. Intraoperative sentinel lymph nodes were not consistent with those in the group (no sentinel lymph node identified was found above one side, and/or frozen pathological metastasis of lymph nodes was found)

### Operation procedures

1. All patients were required to undergo pelvic MRI and upper abdominal CT examination before operation. Patients for whom preoperative endometrial diagnostic curettage pathology suggested endometrioid adenocarcinoma were selected for operation. Informed consent was signed according to the admission and discharge criteria.
2. Indocyanine green was injected into the cervix at 3 o'clock and 9 o'clock positions (Indocyanine green concentration 1.25 mg/ml, 0.5 ml for each site 1–3 mm Superficial injection and 1–2 cm deep injection, 1 ml for each side). SLN was detected by near infrared fluorescence imaging.
3. Operation mode
  Preoperative prophylactic antibiotics should be given at least 30 min before skin cutting;
  In the low lithotomy position, the patient’s arm is parallel to the patient or placed on the patient’s chest;
  Considering the wide variety of instruments available for the operation, the choice of minimally invasive surgical instruments will be decided by the surgeon.
  The minimally invasive abdominal technique and the number of trocar ports used for the operation depend on the judgment of the surgeon.
    Ascites exfoliated cells will be sent for examination. All peritoneal surfaces should be thoroughly examined, including direct examination of the diaphragm. The location of any metastatic disease should be recorded in the surgical report and biopsy should be performed to confirm the diagnosis.
4. Additional instructions
  1. If the suspected enlarged lymph nodes are detected under minimally invasive surgery, then the enlarged lymph nodes are sent to frozen pathology for definite metastasis. The patient will be treated with TH + BSO + PL ± paraaortic lymph node resection, and excluded from the group.
  2. If no sentinel lymph node is found, TH + BSO + PL ± paraaortic lymph node resection will be performed and the patient will be excluded from the group.
  3. If only unilateral sentinel lymph nodes are found, the patient will be excluded from the group and TH + BSO + PL ± paraaortic lymph node resection will be performed.
  4. If there are no suspicious enlarged lymph nodes on either side of the pelvic and abdominal cavity under minimally invasive surgery, and bilateral sentinel lymph nodes are developed, the developed SLN shall be removed and frozen pathology shall be performed during the operation. If the frozen pathology indicates that SLN is negative, they will be randomized into the experimental group or the reference group. Experimental group: SLNB + TH + BSO ± paraaortic lymph node resection; Reference group: SLNB + TH + BSO + PL ± paraaortic lymph node resection. If the frozen pathology of SLN is positive, the patient shall be excluded from the group and treated with TH + BSO + PL ± paraaortic lymph node resection.
  5. Sentinel lymph node ultra-staging will be performed by postoperative pathology: routine Hematoxylin and Eosin (H & E) staining will be performed first. If the result is negative, two adjacent pieces of paraffin at an interval of 5 μm to 50 μm slices will be cut and stained with H & E and cytokeratin AE1/AE3 respectively.
  6. Description of paraaortic lymph node resection: 1) Paraaortic sentinel lymph nodes are developed. The developing lymph nodes are removed and sent to frozen pathology during the operation. If the frozen pathology is negative and the patient has high-risk factors (lymph-vascular space invasion, deep muscle invasion, G3), the paraaortic lymph node resection needs to be done reaching at least the level of the inferior mesenteric artery, and the surgeon will decide whether to reach the level of the renal vein. If the frozen pathology is negative and the patient has no high-risk factors, paraaortic lymph node resection need not be performed. If the frozen pathology is positive, the patient shall be excluded from the group. Paraaortic lymph node dissection shall be performed to reach at least the level of the inferior mesenteric artery, and the surgeon will decide whether to reach the level of the renal vein. 2) No paraaortic sentinel lymph node is developed. Patients without high-risk factors need not undergo paraaortic lymph node resection. Patients with high-risk factors (lymph-vascular space invasion, deep myometrial invasion, G3) need to undergo paraaortic lymph node resection to reach at least the level of the inferior mesenteric artery, and the surgeon will decide whether to reach the level of the renal vein.

### Outcomes

1. Primary Endpoint
  3-year disease-free survival (DFS): Time from patient randomization to disease progression/death.
2. Secondary Endpoint
  Overall survival (OS): Time from randomization to death from any cause.
  Quality of life (QoL): The patient’s quality of life, including the living conditions of patients (EORTC QLQ-C30/CX24).
3. Perioperative clinical data
  Operation time
  Intraoperative blood loss
  Pelvic lymphocyst/Lymphedema of lower limb/Edema of vulva
  / Deep venous thrombosis (DVT)/fever (T ≥ 37.5℃)/Postoperative hospital stay.
4. Sentinel lymph node data
  Identified time of SLN
  Identified ratio of SLN
  Identified site of SLN
  Negative predictive value of SLN
  False negative rate of SLN

### Postoperative adjuvant therapy

This study will assess whether patients need postoperative adjuvant therapy according to National Comprehensive Cancer Network (NCCN) guidelines. The implementation and management of radiotherapy will be carried out according to clinical practice guidelines. Researchers are required to record the dose and start and end time of radiotherapy or concurrent chemoradiotherapy. Isolated tumor cells and micrometastasis can be considered as positive lymph nodes, but they have no impact on the adjuvant treatment of patients. We don’t plan to use molecular typing to guide adjuvant treatment planning since research on adjuvant therapies incorporating molecular classification are currently ongoing and not yet well established by high-level prospective RCT research data, such as RAINBOW, PROTEC IVA, etc. According to the NCCN guidelines, the radiotherapy doctors in the research team develop an adjuvant therapy plan, which is then reviewed by the PI of the sub center, and periodically reviewed by the main PI.

### Qualification certification for participating physicians

The surgeons participating in the operations must be qualified gynecological oncologists and have records to prove that they can well complete minimally invasive TH + BSO + PL ± paraaortic lymph node resection. To minimize the surgical complications of surgeons in the initial learning stage, gynecological oncologists participating in the study must have finished laparoscopy in more than 50 cases of endometrial cancer surgery, with an annual average of 15 cases. Also, laparoscopic ICG fluorescence sentinel lymph node biopsy must have been carried out in more than 30 cases. Also, the trial management committee was provided with non-edited surgical videos from two recent years and the data of 50 surgical patients from three recent years. Case information will be kept confidential to protect the confidentiality of patients. The required information shall include:

1. Age
2. Body mass index
3. Staging
4. Operation time
5. Intraoperative blood loss
6. Length of stay
7. Intraoperative complications
8. Postoperative complications (within 30 days after operation)
9. Blood transfusion volume (intraoperative and postoperative)

The trial management committee will evaluate and judge each surgical video according to the following criteria:

1. surgeon’s surgical techniques
2. anatomical cognition and anatomical ability of pelvic structure
3. surgical techniques to control intraoperative blood loss and prevent intraoperative injury
4. ability to make decisions based on intraoperative exploration
5. accuracy of relevant instruments used during surgery

Once the chief investigator of each center is recognized, he/she can judge the ability of surgeons in his/her institution to cooperate in the study, to ensure that the above criteria are met.

### Randomization and stratification

The subjects were screened by a random system; a random distribution sequence was generated; and the corresponding surgical treatment will be performed according to the random results. Random stratification, age (≥ 50 years or < 50 years), surgical method (laparoscopic surgery or robotic surgery).

### Statistical methods

Disease-free survival and overall survival curves will be evaluated using the Kaplan Meier method. The survival difference between the two groups will be analyzed by log-rank method. The analysis of prognostic factors will use appropriate regression models (such as Cox model). Efficacy indicators will be compared through intention-to-treat analysis including all randomized patients. Toxicity will be analyzed based on the treatment received. The 95% confidence interval of the difference between the two groups will be calculated at the same time. Descriptive statistical analysis will be used for treatment-related adverse events and quality of life scores (QLQ-C30, QLQ-CX24). The normality and equality of differences between the two groups of continuous variables will be evaluated. Discrete variables (such as presence/absence of postoperative infection) will be aggregated by frequency/proportion.

For continuous variables, analysis of variance and/or regression analysis will be used as appropriate. If the assumptions of these tests are violated, an alternative nonparametric test method will be used. Differences in discrete variables between groups will be assessed by chi square test. The risk proportional regression model will be used to explore the prognostic factors such as age, degree of differentiation, lymph node metastasis, and PS-ECOG.

The hypothesis H0: “Sentinel lymph node biopsy alone is not inferior to sentinel lymph node biopsy plus lymphadenectomy for early stage endometrial cancer with negative nodal assessment intraoperatively.”

In the current study, there are three patient groups: intention-to-treat (ITT) group, including all randomized patients who signed the consent form; a group of those in serious violation of the protocol, excluded from the efficacy analysis; and a safe population of all randomized patients who at least start the study treatment.

### Sample size

The primary endpoint of this study is 3-year DFS. According to the results of previous research, patients with stage I endometrial cancer who received laparoscopy had 3-year DFS of 88.6% [18]. It is assumed that the 3-year DFS of the reference group and the experimental group is 88%, and the non-inferior boundary value is 8%. Considering the 20% loss of follow-up rate, 361 subjects need to be included in each group to ensure 80.0% power using a 2-sided log-rank test with a type I error rate of 0.05 to reject the null hypothesis. The total sample size of this study is 722 and the number of outcome events in each group is 40. The sample size calculation was done using the PASS software (version 11.0).

### Visit procedures

Follow-up will be conducted at each center. Written informed consent must be obtained before protocol-related procedures (See Table 1).

**Table 1:** Study procedures and baseline and/or follow-up assessments

	**Inclusion visit**	**Surgery procedure**			**Postoperative Visit**	**Follow-up Visits**
	Up to 30 days to surgery				30 days(± 7 days)	1^st^year-3^rd^ year every 3 month (± 14 days)
**Inform consent**	x		RANDOMIZATION			
**Screening for eligibility**	x					
**Medical history**	x					
**Physical exam**	x				x	x
**Gynecological examination**	x				x	x
**PS-ECOG**	x				x	x
**Tumor assessment**	x				x^b^	x^b^
**Blood examination, liver and renal function, ECG**	x				x	x
**Chest X-ray or Chest CT**						
**Upper abdominal CT**	x					x^c^
**Pelvic MRI**	x					x^c^
**Endometrial diagnostic curettage pathology**	x					
**Surgery approach (laparoscopic or robotic)**		x				
**SLN mapping and biopsy**		Arm A & B				
**Histological examination**		x				
**TH + BSO**		x				
**PLN dissection**				Arm B		
**paraaortic lymph node resection**		x^a^		x^a^		
**Adverse Events (CTCAE V3.0)**					x	x
**Questionnaires (EORTC QLQC30; EORTC QLQ-CX24)**	x					x

*ECG* Electrocardiogram, *PS-ECOG* Performance Status-Eastern Cooperative Oncology Group, *CT* Computed Tomography, *MRI* Magnetic Resonance Imaging

^a^ See “Additional instructions” from “Operation procedures” section

^b^ If necessary

^c^ Upper abdominal CT and pelvic MRI will be done 6 months after the initial treatment, and if necessary within three years

#### Preoperative investigation

Diagnostic curettage of endometrium can be defined as one of the following pathological types: Endometrioid adenocarcinoma; cancerization.

The following examinations are to be completed within 30 days before operation:

1. Chest X-ray or Chest CT examination
2. Upper abdominal CT and Pelvic MRI (PET-CT and/or ultrasound can also be done if there are clinical indications); ECG

The following steps are to be completed 30 days before the operation:

1. Sign written informed consent
2. Record all drugs used currently and in the past 12 weeks (including prescription drugs, over-the-counter drugs, vaccines, vitamins, or proprietary Chinese medicines)
3. Complete the “quality of life” assessment
4. Complete physical and pelvic examinations
5. PS-ECOG
6. Record weight (kg) and height (cm)
7. Perform standard preoperative laboratory examination of each institution
8. Perform serum pregnancy test (if necessary)
9. Perform biochemical tests including creatinine, bilirubin, albumin, alkaline phosphatase (ALP), aspartate aminotransferase (AST), and alanine aminotransferase (ALT)
10. Record whole blood cell count

#### Operative investigation

This visit will perform the following process:

1. Surgery according to random results;
2. Record intraoperative and postoperative information (including operation, histopathological results, intraoperative complications, blood transfusion and other special conditions during operation).

#### Postoperative investigation

The following interviews are planned at 1 month, then every 3 months after surgery for 3 years:

1. Complete the quality of life assessment form (QLQ-C30, QLQ-CX24);
2. Review PS-ECOG;
3. Physical exam and pelvic examination, tumor assessment (if necessary)
4. Record the whole blood cell count and biochemical indexes
5. Adverse event assessment (Common Terminology Criteria for Adverse Events (CTCAE) V3.0);
6. Postoperative pelvic B-ultrasound is used to determine whether there is a lymphocyst. If the patient has symptoms such as swelling and pain in the lower extremities, color Doppler ultrasound in the lower extremities will be used to assess the presence or absence of DVT. The development of lymphedema is investigated and evaluated by measuring the circumference of the legs and combining with the methods of pelvic ultrasound.

In addition, upper abdominal CT and pelvic MRI will be done 6 months after the initial treatment, and if necessary within three years.

### Data confidentiality and storage

The study assistant will collect participants’ relevant clinical data. All participants’ personal data and observation records will be kept confidential. Participants’ identity information will not be disclosed to members outside the research group without obtaining participants’ permission. The identity of participants’ sponsor or study member is confidential. Participants’ files will be kept in a locked filing cabinet for researchers’ reference only. In order to ensure that the research is carried out in accordance with regulations, if necessary, members of the government management department or the ethics review committee can access participants’ personal data in the research unit. This would only be done according to regulations, and they would strictly keep participants’ information secret. Any public report on the results of this research will not disclose any personal information of the participants. Researchers will make every effort to protect the privacy of participants’ personal medical data to the extent permitted by law. If participants do not comply with the study plan, or if there is a study-related injury, or for any other reason, the researchers may terminate participants’ participation in the study.

### Ethics and dissemination

Ethics approval has been obtained for the study protocol by the Institutional Review Board of the Zhejiang Cancer Hospital in Zhejiang, China (Reference number: IRB-2018–235(ke); Date of approval: 18 December 2018). All participants will be given both written and oral information about the study. Patients must sign an informed consent form before being included in the study. Standard treatments will not be affected if the patients refuse to participate in this study. Patients can withdraw at any time.

The Academic and Infection Committee of our hospital will review the safety and effectiveness data collected during the study. This committee will be managed independently of the study and will not participate (directly or indirectly) in the study, and will be responsible for continuously monitoring the following events:

1. Side effects (CTCAE, V3): Grade 3 and 4 adverse events, serious adverse events
2. Patient death events (Level 5)
3. Twice-yearly review of the safety of all data collected during the study

After each meeting, based on the results of the data review, the committee will recommend continuing the research according to the original scheme or will recommend changes to the scheme. In exceptional circumstances, the committee may recommend that the study be discontinued for safety reasons.

The Ethics Committee will conduct a review once a year. According to the results of the data review, the committee will recommend continuing the research according to the original scheme or will recommend changes to the scheme.

## Discussion

Two randomized controlled trials with large samples have proved that lymphadenectomy does not improve the overall recurrence rate and survival rate for patients with endometrial cancer. On the contrary, it increases the incidence of complications and even mortality [1, 2]. So far, there is still a lack of strong evidence as to whether sentinel lymph node biopsy can replace lymphadenectomy for early endometrial cancer. ICG combined with near-infrared fluorescence imaging is a new tracer method for lymphatic detection and it has been proved to be superior to ^99^Tc, blue dye, and other methods. A systematic review showed that the total detection rate of SLN detected by ICG combined with near-infrared fluorescence imaging was 94%, which was significantly higher than that by blue staining (86%) and radionuclide method (86%). The total sensitivity of this technique for detecting SLN is 94% and the negative prediction rate is 100%. ICG combined with near-infrared fluorescence imaging can prevent patients with early endometrial cancer from unnecessary lymphadenectomy, and can reduce complications such as long operation time, bleeding, pelvic lymphocyst, chylous ascites, and infection. To some extent, it can reduce the mortality, reduce the length of hospital stay, and reduce medical expenses, thereby benefitting patients.

Naturally, there is a certain difference between surgical trials and drug trials. Surgical trials always face difficulties because the quality is related not only to the design and conduct of trials, but also to the surgery itself. These deviations come from randomization of surgeon/patient selection. In the present study, the qualifications and experience of surgeons may have some deviation. Therefore, we will need more experiments and trials to further verify the results.

### Trial status

This study was approved by the Institutional Review Board of the Zhejiang Cancer Hospital in Zhejiang, China, in 18 December 2018. The trial was registered on chictr.org.cn and a registration number was obtained. Recruitment of participants started in January 2019. The last participant will be expected to achieve the primary endpoint (3-year disease-free survival) in 2028. The protocol version number was 5.0 and the protocol version date was 16 Jun 2022.

## Data Availability

The datasets used and/or analyzed during the current study are available from the corresponding author on reasonable request.

## Abbreviations

EC: Endometrial cancer
SLN: Sentinel lymph node
ICG: Indocyanine green
SLNB: Sentinel lymph node biopsy
TH: Total hysterectomy
BSO: Bilateral salpingo-oophorectomy
PL: Pelvic lymphadenectomy
MRI: Magnetic Resonance Imaging
CT: Computed Tomography
RECIST: Response Evaluation Criteria in Solid Tumors
PS-ECOG: Performance Status-Eastern Cooperative Oncology Group
H & E: Hematoxylin and Eosin
ECG: Electrocardiogram
DFS: Disease-free survival
OS: Overall survival
QoL: Quality of life
QLQ: Quality of Life Questionnaire
EORTC: European Organization for Research and Treatment of Cancer
DVT: Deep venous thrombosis
NCCN: National Comprehensive Cancer Network
ITT: Intention-to-treat
PET/CT: Positron Emission Tomography/ Computed Tomography
ALP: Alkaline phosphatase
AST: Aspartate aminotransferase
ALT: Alanine aminotransferase
